# Design and evaluation of nano-hydroxyapatite/poly(vinyl alcohol) hydrogels coated with poly(lactic-co-glycolic acid)/nano-hydroxyapatite/poly(vinyl alcohol) scaffolds for cartilage repair

**DOI:** 10.1186/s13018-019-1450-0

**Published:** 2019-12-17

**Authors:** Weiping Su, Yihe Hu, Min Zeng, Mingqing Li, Shaoru Lin, Yangying Zhou, Jie Xie

**Affiliations:** 1 Department of Orthopedics, Xiangya Hospital, Central South University, No.87 Xiangya Road, Changsha, 410008 Hunan China; 2 Department of Oncology, Xiangya Hospital, Central South University, No.87 Xiangya Road, Changsha, 410008 Hunan China

**Keywords:** Poly(lactic-co-glycolic acid), Poly(vinyl alcohol), Nano-hydroxyapatite, Tissue engineering

## Abstract

**Background:**

Poly(vinyl alcohol) (PVA) hydrogels have been widely used in synthetic cartilage materials. However, limitations of PVA hydrogels such as poor biomechanics and limited cell ingrowth remain challenges in this field.

**Methods:**

This work aimed to design novel nano-hydroxyapatite (nano-HA)/poly(vinyl alcohol) (PVA) hydrogels coated with a poly(lactic-co-glycolic acid) (PLGA)/nano-HA/PVA scaffold to counter the limitations of PVA hydrogels. The core, comprising nano-HA/PVA hydrogel, had the primary role of bearing the mechanical load. The peripheral structure, composed of PLGA/nano-HA/PVA, was designed to favor interaction with surrounding cartilage.

**Results:**

The double-layer HA/PVA hydrogel coated with PLGA/HA/PVA scaffold was successfully prepared using a two-step molding method, and the mechanical properties and biocompatibility were characterized. The mechanical properties of the novel PLGA/HA/PVA scaffold modified HA/PVA hydrogel were similar to those of native cartilage and showed greater sensitivity to compressive stress than to tensile stress. Rabbit chondrocytes were seeded in the composites to assess the biocompatibility and practicability in vitro. The results showed that the peripheral component comprising 30 wt% PLGA/5 wt% HA/15 wt% PVA was most conducive to rabbit chondrocyte adhesion and proliferation.

**Conclusions:**

The study indicated that the double-layer HA/PVA hydrogel coated with PLGA/HA/PVA scaffold has the potential for cartilage repair.

## Introduction

A cartilage defect is a common clinical problem with cartilage degeneration. It is very difficult to treat in the clinic [1]. Cartilage tissue has a low regenerative capacity owing to its poor blood supply, particular biomechanics, and complex structure [2, 3]. Various approaches to improving cartilage regeneration have been investigated, such as osteochondral allografts, microfracture, osteoarticular transfer systems, and autologous chondrocyte implantation. However, these procedures usually result in the formation of non-hyaline cartilage with inferior long-term results [3–6]. Hydrogel-based biomaterials possess similar microstructure to that of natural cartilage and have shown significant potential in the field of cartilage repair [7–11]. Poly(vinyl alcohol) (PVA) hydrogel, in particular, is widely studied owing to its advantageous physicochemical properties and useful biomechanical properties (their compressive and elastic mechanical properties) [12]. The PVA hydrogel is a hydrophilic material with a three-dimensional network structure, and its pore size is on the order of several micrometers to several tens of micrometers similar to articular cartilage. It contains a large amount of water and is permeable, which can provide lubrication to the joint surface and avoid wear debris [13, 14].

However, the poor mechanical strength and durability, as well as bioactivity, of hydrogels limit their further application [15, 16]. Currently, the main complication of the potential clinical application of PVA is that PVA hydrogel is not suitable for cell attachment and proliferation [17, 18]. In recent years, the composition scaffold has attracted a lot of interests [19–21]. Many studies have shown that some materials such as poly(lactic-co-glycolic acid) (PLGA), and nano-hydroxyapatite (nano-HA) can be used to improve the cellular affinity and biomechanics of PVA hydrogel [7, 22–26]. The objective of this study was to construct a novel double-layer artificial hydrogel consisting of core and peripheral components. The primary role of the core, composed of nano-HA/PVA hydrogel, was to bear the mechanical load, and the peripheral structure, comprising PLGA/nano-HA/PVA, was designed to favor interaction with surrounding cartilage. Thus, addressing the limitations of PVA hydrogel, a novel double-layer hydrogel with good biocompatibility and excellent mechanical properties was fabricated. Besides, the morphological characteristics, mechanical features, and biocompatibility of the double-layer nano-HA/PVA hydrogel coated with a PLGA/HA/PVA scaffold were described.

## Materials and methods

### Fabrication, viability, and biocompatibility of the peripheral structure (PLGA/nano-HA/PVA hydrogel)

#### Hydrogel preparation

The PLGA/HA/PVA hydrogel was fabricated based on our previous method using the solvent extraction, evaporation technique, and repeated freeze-thaw cycling method [27]. First, PVA (341,584, 99+% hydrolyzed, M_w_ 89,000–98,000, Sigma, USA) and nano-HA (677,418, nanopowder, < 200 nm particle size (BET), ≥ 97%, synthetic, Sigma, USA) were incorporated into double-distilled water. The mixture was then heated to 90 °C for 90 min in a water bath with thermostatic magnetic mixer stirring. PLGA (lactide: glycolide 50:50, ester terminated, M_w_ 38,000–54,000, Sigma, USA) was dissolved in dichloromethane with ultrasonic stirring, which created a primary emulsion, then the primary emulsion was added to the PVA-nano-HA mixture. The PLGA/nano-HA/PVA solution was stirred with a thermostatic magnetic mixer to evaporate the dichloromethane, and then the solution was carefully injected into a mold. Finally, the method for crosslinking is freeze-thaw [28] which mold was frozen at − 20 °C for 21 h and allowed to thaw for 3 h at room temperature. The freeze-thaw cycle was repeated five times to increase the density of crosslinking.

#### Viability and biocompatibility of the peripheral structure (PLGA/nano-HA/PVA hydrogel)

Based on microscopic morphology we have reported previously [27], we selected groups with PVA mass fraction of 5 wt%(E1), 10 wt%(E2), or 15 wt%(E3); PLGA of 30 wt%; and nano-HA of 5 wt% for investigation. The control group contained HA: 5 wt%, PVA: 15 wt% (CG).

The animal study was approved by the Medical Ethics Committee of Xiangya Hospital Central South University. Four-week-old white New Zealand rabbits were euthanized by air embolism. Under aseptic conditions, the articular cartilage was then collected from the rabbit hip, knee, and shoulder joints and sliced into approximately 1 × 1 × 1 mm^3^ sections. The cartilage fragments were washed with PBS solution three times before being digested in 0.2% collagenase type-II at 37 °C for 6 h. The supernatant was then transferred to a new tube and centrifuged at 300 g for 5 min to collect the cell pellets. The cells were cultured in DMEM medium containing 1% penicillin/streptomycin and 10% fetal bovine serum at 37 °C in a humidified incubator containing 5% CO_2_. When the cells reached 80–90% confluence, they were collected and adjusted to a concentration of 1 × 10^6^/mL. The cells were observed with microscopy and adapted to grow in culture. Also, the chondrocytes were identified with hematoxylin-eosin, toluidine blue staining, and type II collagen immunohistochemistry.

The scaffold (5 × 5 × 5 mm^3^) was freeze-dried using a lyophilizer (VFD-1000, Biocool, China) and sterilized with ethylene oxide. A set volume of cell suspension (1 × 10^6^/100 μL) was dropped on the scaffolds (20 scaffolds per group; five for cell adhesion test, five for MTT assay, five for Western blot analysis of glycosaminoglycan and collagen type II, and five for HE, toluidine blue, and immunohistochemistry staining) in a 24-well cell culture plate. The complexes were incubated at 37 °C in a 5% CO_2_ humidified incubator for 2 h, then 2 mL of fresh medium (pre-warmed to 37 °C) was carefully added to each well and incubation was continued. After the cells were inoculated with the scaffold material for 24 h, the co-cultured scaffolds were taken out, and non-adhered cells on the scaffold were eluted with PBS solution. The eluate and the culture medium of each group were collected, and the cells were counted by centrifugation. The cell adhesion rate was calculated based on the method described before [27].

MTT assay was used to evaluate cell proliferation. The growth of chondrocytes in each group was analyzed by HE, toluidine blue, and immunohistochemistry staining of collagen type II after co-culture for 3, 7, and 14 days. Total cell protein was then extracted from each group using a cell lysis solution (Cell Signaling Technology, USA). The expression of glycosaminoglycan and collagen type II were quantitatively determined by Western blotting. Western blot analysis of cell lysates was performed as described [29].

### Fabrication and evaluation of double-layer PVA hydrogel articular cartilage

#### Fabrication of the double-layer PVA hydrogel articular cartilage

Based on the morphological characteristics and practicability of the PLGA/HA/PVA hydrogels, we chose the PLGA: 30 wt%, HA: 5 wt%, PVA: 15 wt% hydrogel as the peripheral component. For the core, the proportion of components was nano-HA: 5 wt% and PVA: 20 wt%.

**Fig. 1:**
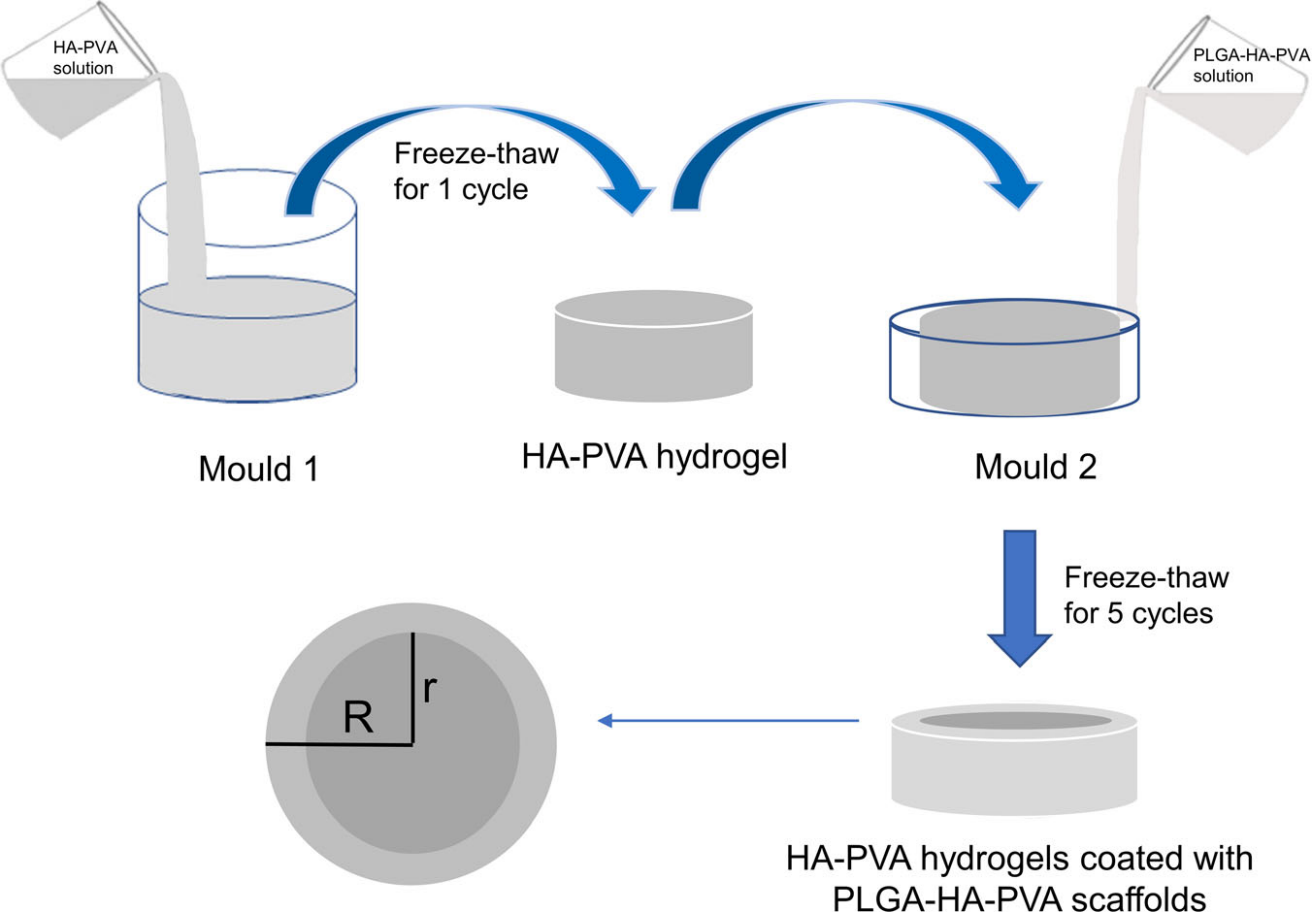
HA-PVA hydrogels coated with PLGA-HA-PVA scaffolds was prepared by two-step molding

A novel HA/PVA hydrogel modified with a PLGA/HA/PVA scaffold was prepared using two-step molding. PVA and nano-HA were incorporated into double-distilled water. The mixture was heated to 90 °C for 90 min in a water bath with thermostatic magnetic mixer stirring. The mixture was then injected into mold 1 (radius r), frozen for 21 h at − 20 °C, and allowed to thaw for 3 h at room temperature to form the core. The PLGA/HA/PVA solution was then injected into mold 2 (radius R), as described above, which was larger than mold 1, frozen for 21 h at − 20 °C and allowed to thaw for 3 h at room temperature to give the peripheral section. The freeze-thaw cycle was repeated five times to increase crosslinking in the transition zone between the two hydrogels (Fig. 1). Through control of the bottom surface size (*r*^2^, *R*^2^) of the two molds, we prepared groups of HA/PVA hydrogel modified with PLGA/HA/PVA scaffold with different constituent ratios. Components that contained HA/PVA hydrogel (control group one, CG1) or PLGA/HA/PVA hydrogel (control group two, CG2) were used as controls. The radius of mold 2 was 10 mm, and that of mold 1 was 7 mm in experimental group one (EG1), 8 mm in experimental group two (EG2), and 9 mm in experimental group three (EG3).

#### Constituent ratio, ESEM detection, and mechanical features of the composites

A bottom-emitting image was obtained in a dark room, and the constituent ratio of each section was calculated using ImageJ software. Also, the microstructure of the composites was observed by ESEM. The mechanical properties of the composites, including compressive and tensile tests, were evaluated using a universal mechanical testing machine (DDL100, Changchun, China). The sample (20 mm × 20 mm) was cylindrical and was directly placed on the test bench for pressurization. The stress/strain rate for the test was 5 mm/min. For the compressive mechanical properties test, the compression was stopped when the compression strain reaches 60%. For the tensile mechanical properties test, we stuck the end of the material with the aluminum alloy. After 24 h, the adhesion reached the maximum strength and was tested on the machine. The stress/strain rate for the test was 5 mm/min, and the test index was the stress-strain curve and strength of the material. Three samples in each group were selected, and each sample was measured three times.

### Statistical analysis

All values are reported as mean ± standard deviation (SD). When normal distribution and homogeneity of variance were obeyed, statistical analysis of differences between groups was evaluated using one-way ANOVA, and the pairwise comparison among the means was carried out using the LSD method. In addition, Tamhane’s T2 test was used for the unequal variances. Using SPSS19.0 statistical software, statistical significance was defined as *P* < 0.05.

## Results and discussion

### Biocompatibility of PLGA/nano-HA/PVA hydrogels

#### Cellular adhesion and proliferation

The morphology and growth features of chondrocytes were as follows. On the first day after resuscitation, the cultured cells grew on the wall of the flask with spindle-shaped morphology. On day 7, cells were spindle-shaped and whorled or parallelled along the longitudinal axis; toluidine blue staining led to cells being stained light blue and the nucleolus being stained purple-blue (Additional file 1: Figure S1); immunohistochemical staining of type II collagen (Additional file 1: Figure S1) showed yellow-brown granules in the cytoplasm of the chondrocytes, and the nucleus was stained blue. The results of staining showed that chondrocytes cultured in vitro had type II collagen expression in the cytoplasm and cell membrane. The cell growth curve (Additional file 1: Figure S1) showed that the proliferation of chondrocytes increased significantly after 3 days of culture, reached a peak on day 9, and began to decline from day 10. The second-generation chondrocytes isolated and cultured showed the best growth. As the number of passages increased, the rate of proliferation declined.

**Fig. 2:**
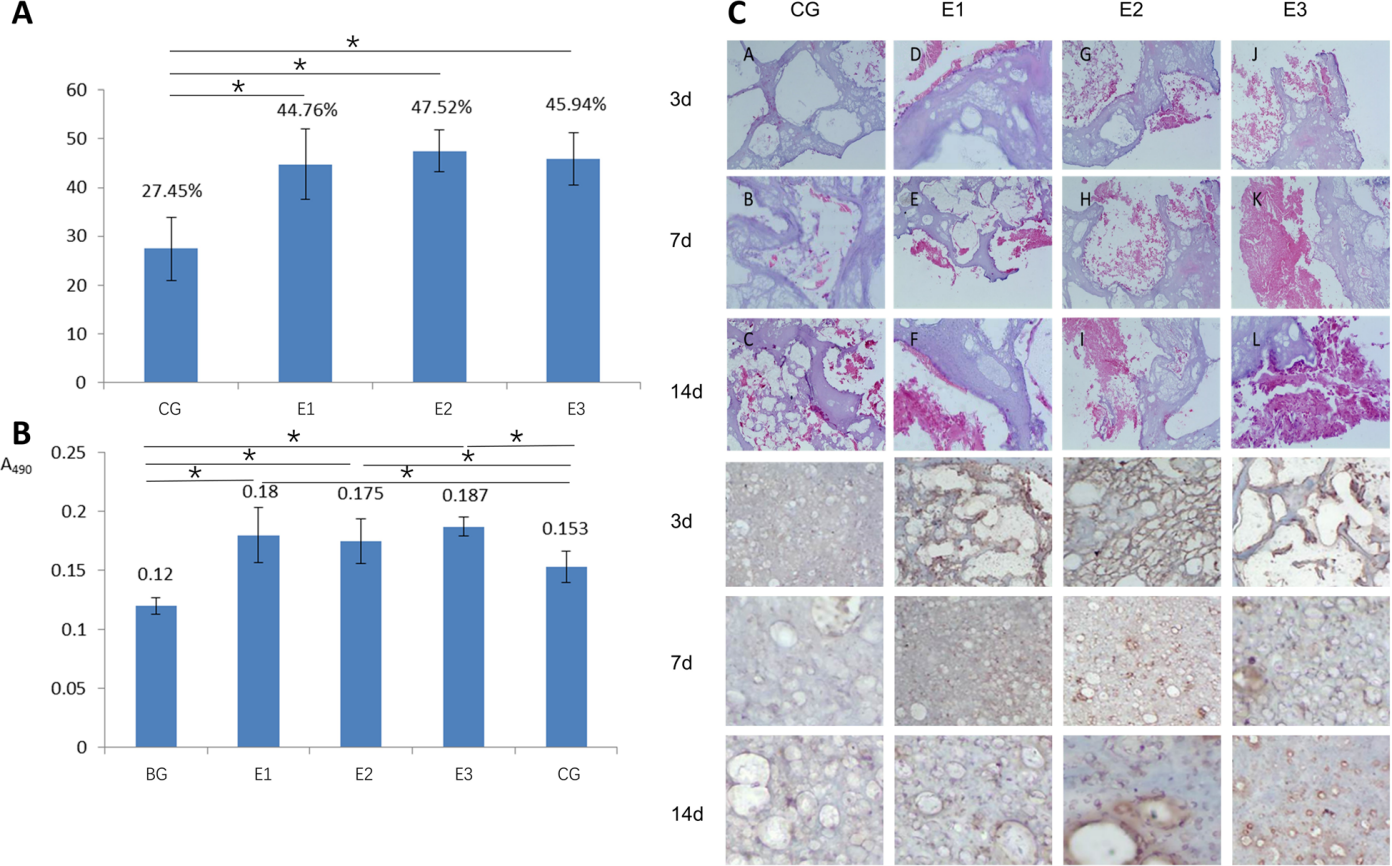
The results of cellular adhesion ability (**a**). The results of cellular proliferation by MTT (**b**). HE staining and immunohistochemical staining of type II collagen (**c**) after co-culturing rabbit chondrocytes with scaffolds for 3 days, 7 days, 14 days; PVA mass fraction of 5 wt% (E1), 10 wt% (E2), or 15 wt% (E3); PLGA of 30 wt%; and nano-HA of 5 wt% were used for investigation. The control group contained HA: 5 wt%, PVA: 165 wt% (CG)

The results of cellular adhesion are shown in Fig. 2. The adhesion in the experimental groups was significantly higher than that in the control group (*P* < 0.05), while there were no statistical differences among the three experimental groups (*P* > 0.05). This result shows that the addition of PLGA significantly favored the adhesion of chondrocytes. As described above, the hydrophobic PLGA particles and nano-HA facilitated integration with surrounding cartilage and improved the porosity of the scaffolds. The cellular attachment significantly increased as a result of the combined effect of PLGA and nano-HA. Since the amount of PLGA and nano-HA were the same, there was almost no difference between the three experimental groups.

The results of cell proliferation are shown in Fig. 2. The proliferation in the groups with scaffolds was enhanced compared with the control group (*P* < 0.05); in addition, the proliferation of the experimental groups was higher than that for the control group (*P* < 0.05) and there was no statistical difference between the three experimental groups (*P* > 0.05). Some researchers have shown that the morphology and mechanical properties of scaffolds, such as the elastic modulus, affect how seeded cells behave [30, 31]. The results of this study showed that the presence of scaffolds helps the proliferation of cells. Furthermore, the PLGA/PVA/nano-HA hydrogels performed better than the PVA/nano-HA hydrogels. The reason for this observation might be that the elastic modulus of the PLGA/PVA/nano-HA hydrogels was more conducive to proliferation than that of the PVA/nano-HA hydrogels. The results of the tests, therefore, showed that the addition of PLGA microparticles favored the adhesion and proliferation of seeded chondrocytes.

#### Histological and immunohistochemical staining of PLGA/nano-HA/PVA hydrogels co-cultured with chondrocytes

HE staining showed numerous cells in the pores of the hydrogels (Fig. 2). Cell proliferation of the experimental groups (EG1, EG2, EG3) was found to be enhanced compared with the control group and was positively correlated with the time of co-culture. In addition, the amount of matrix secreted by chondrocytes in the experimental group was more than in the control group. Among the experimental groups (EG1, EG2, EG3), proliferation of the EG3 group was the most pronounced, and the extracellular matrix was clearly connected.

Results of toluidine blue staining were similar to those of HE staining (Additional file 1: Figure S3). The toluidine blue staining was positive after 3, 7, and 14 days of co-culture in the experimental groups (EG1, EG2, EG3) and control group (CG). Cell proliferation of the experimental groups (EG1, EG2, EG3) was observed to be more active than for the control group and was positively correlated with the time of co-culture. Among the experimental groups (EG1, EG2, EG3), the proliferation of the EG3 group was the most pronounced.

Immunohistochemical staining of type II collagen (Fig. 2) was positive in both the experimental groups (EG1, EG2, EG3) and control group (CG) after 3, 7, and 14 days of co-culture. In the experimental groups (EG1, EG2, EG3), the staining results were strongly positive. The matrix was brownish yellow, and coarse collagen fibers could be seen. The expression of type II collagen was positively correlated with the time of co-culture. Among the experimental groups, the expression of type II collagen was most pronounced in the EG3 group.

The Western blot results showed that the expression of COL2 and GAG could be detected in both the experimental and control groups (Additional file 1: Figure S2). The relative expression of COL2 and GAG increased over time (*P* < 0.05). After co-culturing for 3, 7, and 14 days, expression of COL2 and GAG in the experimental groups was higher than for the control group (*P* < 0.05), while the expression in group C was higher than for the other two groups (*P* < 0.05).

This finding supported the observation that the PLGA/PVA/nano-HA hydrogels performed better in improving cell proliferation and secretion of chondrogenic matrix compared with PVA/nano-HA hydrogels. The improvement shown by the hydrogel is thought to be related to the addition of PLGA microparticles, which improved the biocompatibility of the scaffolds [32, 33]. Meanwhile among the experimental group, EG3 (5% HA, 30% PLGA, 15% PVA) showed greater cell proliferation and higher expression of COL2 and GAG than the other two experimental groups. However, the pore size and porosity in EG3 were the smallest and lowest, respectively, among the experimental groups. This indicated that chondrocyte proliferation and secretion of the chondrogenic matrix was not influenced by pore size and porosity alone [34]. It is reported that the elastic modulus of a scaffold plays a role in the differentiation of stem cells [35]. Whether the elastic modulus has the same effect on chondrocytes remains a question. Based on our results, as the PVA content increased, the material became denser, and the modulus of elasticity increased, leading to better chondrocyte proliferation and secretion of the chondrogenic matrix. Therefore, we conclude that the elastic modulus, pore size, and porosity all influenced chondrocyte proliferation. More research is needed to explore the most suitable elastic modulus, pore size, and porosity for this composite.

### Morphological characterization of the double-layer HA/PVA hydrogel coated with PLGA/HA/PVA scaffold

A novel HA/PVA hydrogel modified with PLGA/HA/PVA hydrogel was successfully prepared. The components were molded into a cylinder-like shape with a smooth surface. The peripheral component consisting of PLGA/HA/PVA hydrogel was securely bound to the HA/PVA hydrogel core and the cross-connecting interface between the peripheral and core sections was observed. Based on the bottom-emitting image obtained, the constituent ratio of the different sections was calculated (Additional file 1: Figure S4). The constituent ratio of the core section was reduced compared with the original mold 1. This indicated that hydrogels at the periphery of the core zone combined with the hydrogel in the peripheral section during preparation to form the cross-connecting zone. The constituent ratio of the cross-connecting section decreased when *r*^2^/*R*^2^ increased. The reason for this might be that when *r*^2^/*R*^2^ increased, the volume of PLGA/HA/PVA solution added was reduced, resulting in a smaller amount of dissolved HA/PVA hydrogel in the core region.

**Fig. 3:**
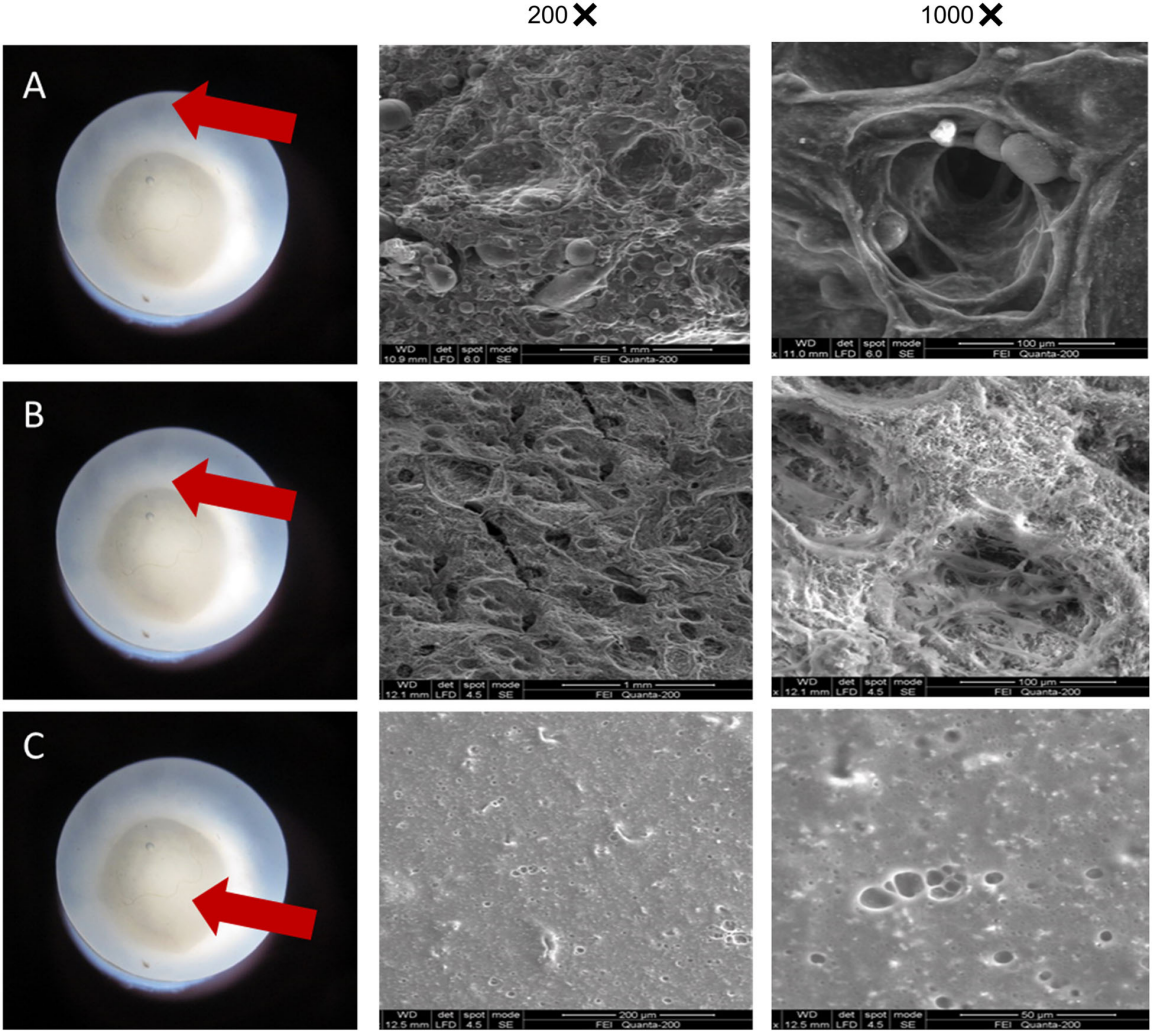
The SEM images of the core (**c**), cross-connecting section (**b**), and peripheral section (**a**)

The SEM images are shown in Fig. 3. We detected that the core, cross-connecting section, and peripheral section were all porous structures. In the peripheral section, the pores were distinct and ranged in size from tens to hundreds of micrometers. PLGA microspheres were attached to the edges of the pores. The diameter of the PLGA microspheres ranged from 30 to 40 μm. In the cross-connecting section, the porosity of the structure was lower than for the peripheral section and the pore size was in the tens of micrometers range. In the core, the pore size was approximately tens of micrometers and no PLGA microspheres were detected.

**Fig. 4:**
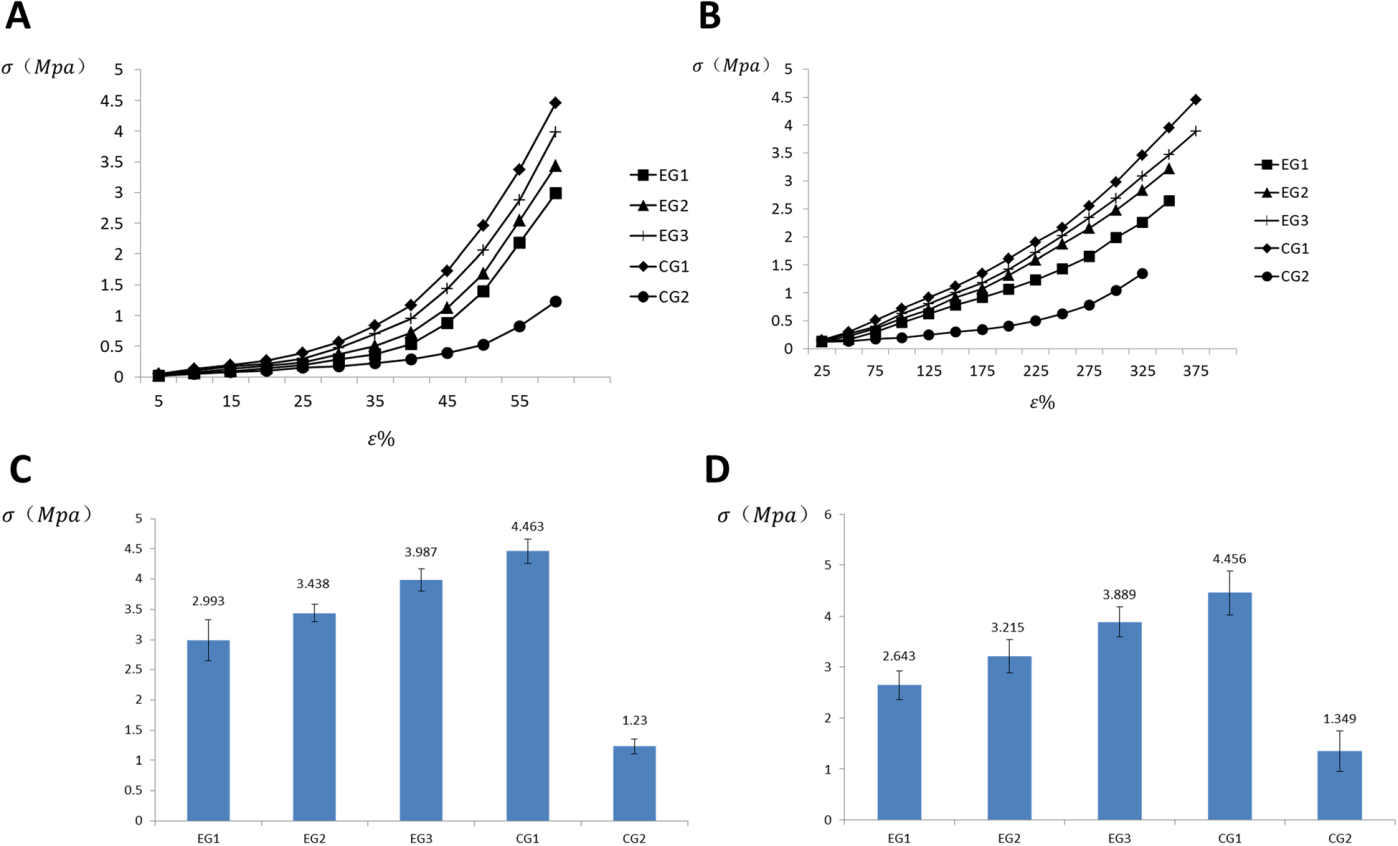
The stress and strain cerves (**a**). The tensile stress and strain curves (**b**). The ultimate compressive stress of each group (**c**). The ultimate tensile stess of each group (**d**)

The compressive stress-strain curves are shown in Fig. 4. The strain of HA/PVA hydrogel modified with PLGA/HA/PVA scaffold prepared in this study was nonlinear with the change of compressive stress and showed an exponential fit indicating that the hydrogel that we prepared was a viscoelastic material. When the hydrogels in each group were at the same strain, the PLGA/HA/PVA hydrogel received minimal compressive stress; the PLGA/HA/PVA scaffold-modified HA-PVA hydrogel received more compressive stress compared with the PLGA/HA/PVA hydrogel, and the stress increased increasing composition ratio of the HA/PVA hydrogel. The higher the strain, the greater the difference that was observed. The compressive stress of HA/PVA alone was the highest among the groups.

The ultimate compressive stress of each group is shown in Fig. 4. The ultimate compressive stress of the PLGA/HA/PVA scaffold-modified HA/PVA hydrogel in each experimental group (EG1, EG2, EG3) was lower than that of the CG1 group (HA/PVA hydrogel only) (*P* < 0.05); however, all were higher than those of the PLGA/HA/PVA hydrogel in the CG2 group (*P* < 0.05). Among the experimental group, the ultimate compressive stress of the hydrogel increased with the increasing composition ratio of HA/PVA (*P* < 0.05).

The tensile stress and strain curves are shown in Fig. 4. The strain of each PLGA-HA-PVA scaffold-modified HA-PVA hydrogel prepared in this study was nonlinear with the change of tensile stress. When the materials in each group were at the same strain, the tensile stress of the PLGA/HA/PVA hydrogel (CG2) was the lowest. The tensile stress of each PLGA/HA/PVA scaffold-modified HA/PVA hydrogel group (EG1, EG2, EG3) was lower than that of the HA/PVA hydrogel (CG1), which withstood the greatest tensile stress. Among the experimental group, the tensile stress increased with increasing HA/PVA hydrogel composition ratio.

The ultimate tensile stress of each group is shown in Fig. 4. The ultimate tensile stress of each PLGA/HA/PVA scaffold-modified HA/PVA hydrogel in the experimental group (EG1, EG2, EG3) was lower than that of the CG1 group (HA-PVA hydrogel) (*P* < 0.05); however, all were higher than that of the PLGA/HA/PVA hydrogel in the CG2 group (*P* < 0.05). Among the experimental group, the ultimate tensile stress of the material increased with the increase of HA-PVA composition ratio (*P* < 0.05).

A HA/PVA hydrogel modified with PLGA/HA/PVA scaffold can be successfully prepared by two-step molding. Based on the above results, HA/PVA hydrogel modified with PLGA/HA/PVA scaffold is a viscoelastic material. The compressive stress-strain of each experimental group showed an exponential rate of change, while the tensile stress-strain showed an increasing polynomial trend, indicating that the PLGA/HA/PVA scaffold-modified HA/PVA hydrogel is more sensitive to compressive stress than tensile stress and has similar mechanical properties to natural cartilage tissue [36, 37]. Because the ultimate stress of the HA/PVA hydrogel is significantly higher than that of the PLGA/HA/PVA scaffold, the higher the composition ratio of HA/PVA in each experimental group, the greater the ultimate stress of the material and the better the biomechanical properties. Using the two-step formation method to prepare this new type of artificial cartilage, the composition ratio of the material can be determined, allowing the biomechanical properties of the composite material to be controlled by adjusting the surface area ratio of mold 1 and mold 2 (*r*^2^/*R*^2^) during the preparation process.

## Conclusions

Structurally stable HA/PVA hydrogel modified with a PLGA/HA/PVA scaffold can be successfully prepared using a two-step formation method to give a smooth surfaced material. It can be divided into a peripheral section, consisting of PLGA/HA/PVA scaffold, core section, consisting of HA/PVA hydrogel, and a cross-connecting interface. The peripheral section retains the characteristics of the PLGA/HA/PVA scaffold, which promotes the adhesion and proliferation of chondrocytes in vitro culture. The core section retains the characteristics of HA/PVA, which has good mechanical properties. This new material is a typical viscoelastic material exhibiting biomechanical properties similar to cartilage and is more sensitive to compressive stress than tensile stress. The biomechanical properties of this new hydrogel can be controlled to meet the physical requirements by adjusting the surface area ratio of mold 1 and mold 2 (*r*^2^/*R*^2^) during the preparation process.

## Supplementary information

**Additional file 1: Figure S1.** Toluidine blue staining(A) and immunohistochemical staining of type II collagen(B) of chondrocytes; The growth curve of rabbit chondrocytes(C). G represents the cell-generation of rabbit chondrocytes passage number. **Figure S2.** Western-Blot analysis of Col2 and GAG protein expression and actin expression after co-culturing rabbit chondrocytes with scaffold for 3d,7d,14d. PVA mass fraction of 5wt%(E1), 10wt%(E2), or 15wt%(E3); PLGA of 30wt%; and nano-HA of 5wt% was used for investigation. The control group contained HA: 5wt%, PVA: 15wt% (CG). **Figure S3.** Results of toluidine blue staining after co-culturing rabbit chondrocytes with scaffold for 3d,7d,14d; PVA mass fraction of 5wt%(E1), 10wt%(E2), or 15wt%(E3); PLGA of 30wt%; and nano-HA of 5wt% were used for investigation. The control group contained HA: 5wt%, PVA: 15wt% (CG). **Figure S4.** The bottom-emitting image of the double-layer HA/PVA hydrogel coated with PLGA/HA/PVA scaffold. The radius of mold 2 was 10 mm, and that of mold 1 was 7 mm in experimental group one (EG1), 8 mm in experimental group two (EG2), and 9 mm in experimental group three (EG3).

## Data Availability

The datasets used and analyzed during the current study are available from the corresponding author on reasonable request.

## Abbreviations

HA: Hydroxyapatite
PLGA: Poly(lactic-co-glycolic acid)
PVA: Poly(vinyl alcohol)
